# Emerging Minimally Invasive Technologies for the Detection of Skin Cancer

**DOI:** 10.3390/jpm11100951

**Published:** 2021-09-24

**Authors:** Joon Min Jung, Ji Young Cho, Woo Jin Lee, Sung Eun Chang, Mi Woo Lee, Chong Hyun Won

**Affiliations:** 1 Department of Dermatology, Asan Medical Center, University of Ulsan College of Medicine, Seoul 05505, Korea; bban29@hanmail.net (J.M.J.); uucm79@hanmail.net (W.J.L.); changse2016@gmail.com (S.E.C.); miumiu@amc.seoul.kr (M.W.L.); 2 Department of Medical Science, Asan Medical Institute of Convergence Science and Technology, Asan Medical Center, University of Ulsan College of Medicine, 88 Olympic-ro 43-gil, Songpa-gu, Seoul 05505, Korea; y0ncjy@naver.com

**Keywords:** skin cancer, basal cell carcinoma, squamous cell carcinoma, melanoma, reflectance confocal microscopy, optical coherence tomography, high-frequency ultrasound, electrical impedance spectroscopy, pigmented lesion assay, Raman spectroscopy

## Abstract

With the increasing incidence of skin cancer, many noninvasive technologies to detect its presence have been developed. This review focuses on reflectance confocal microscopy (RCM), optical coherence tomography (OCT), high-frequency ultrasound (HFUS), electrical impedance spectroscopy (EIS), pigmented lesion assay (PLA), and Raman spectroscopy (RS) and discusses the basic principle, clinical applications, advantages, and disadvantages of each technology. RCM provides high cellular resolution and has high sensitivity and specificity for the diagnosis of skin cancer. OCT provides lower resolution than RCM, although its evaluable depth is deeper than that of RCM. RCM and OCT may be useful in reducing the number of unnecessary biopsies, evaluating the tumor margin, and monitoring treatment response. HFUS can be mainly used to delineate tumor depths or margins and monitor the treatment response. EIS provides high sensitivity but low specificity for the diagnosis of skin malignancies. PLA, which is based on the genetic information of lesions, is applicable for the detection of melanoma with high sensitivity and moderate-to-high specificity. RS showed high accuracy for the diagnosis of skin cancer, although more clinical studies are required. Advances in these technologies for the diagnosis of skin cancer can lead to the realization of optimized and individualized treatments.

## 1. Introduction

Skin cancer is by far the most common type of cancer. Globally, its incidence has increased over the past decades, with more than 1.5 million cases, including non-melanoma skin cancers (NMSCs) and melanomas newly diagnosed in 2020 [1]. Its resultant economic burden and impact on patients’ health have been enormous. An accurate and early diagnosis of suspicious skin lesions is very important for the personalized management of this disease. As with other internal malignancies, the morbidity and mortality of skin cancer can be minimized with early detection. This is particularly true for malignant melanoma, which is a potentially aggressive cancer with a high tendency for lymphatic and visceral metastasis [2,3]. The 5-year survival rate of early melanoma is 99%, whereas that of metastasized melanoma decreases to 20% [4]. The gold standard approach for the diagnosis of skin cancer is histopathological examination after lesional biopsy. However, the procedure is invasive and is associated with a risk of permanent scar formation. Considering that different types of skin cancer can develop in the cosmetically sensitive head and neck regions, this potential morbidity cannot be ignored. In addition, diagnosis through histopathological examination is not always definitive, with a discordance rate of 14–75% among dermatopathologists for the diagnosis of pigmented skin lesions [5,6]. The diagnostic discordance is especially pronounced in the case of dysplastic nevi and melanoma in situ [6]. To minimize the frequency of invasive biopsy procedures and improve the diagnostic accuracy of skin cancer, many emerging noninvasive technologies have been developed as an alternative to skin biopsy. These technologies enable screening and sequential follow-up of multiple suspicious lesions without frequent biopsies, which is especially beneficial for patients with multiple atypical nevi or field cancerization. They have ushered in a new era of skin cancer diagnosis as they are combined with machine-based learning along with the development of computer science [7]. However, for their application to clinical practice, there are still some obstacles to be overcome, such as diagnostic accuracy, time, and expenses. This review discusses the utility and limitations of these technologies for the detection of skin cancer, focusing on reflectance confocal microscopy (RCM), optical coherence tomography (OCT), high-frequency ultrasound (HFUS), electrical impedance spectroscopy (EIS), pigmented lesion assay (PLA), and Raman spectroscopy (RS).

## 2. Reflectance Confocal Microscopy

RCM is a noninvasive in vivo imaging tool that provides cellular resolution similar to that of histological examination [8]. Since its introduction, it has been widely used for the detection of skin cancer, mapping of tumor margins before surgical intervention, evaluation of treatment response, and surveillance of tumor recurrence [9]. It has been approved by the US Food and Drug Administration for processing images of different types of cutaneous cancer for the purpose of decreasing the frequency of unnecessary and invasive biopsy procedures. Wide-probe RCM must be fixed on the target lesion on flat skin surfaces and can create up to 8 × 8-mm^2^-sized mosaic images [8]. Handheld RCM, on the other hand, can cover only a 0.75 × 0.75-mm^2^-sized area [10,11]. However, it does not require fixation on the skin, which makes it suitable for assessing lesions located on curved areas such as the ears. The handheld version has a shorter imaging time than wide-probe RCM [10]. While RCM has the potential to be applied to all types of skin cancer with high sensitivity and specificity because of its high resolution, the restricted depth of penetration can lead to false-negative results for tumors located below the papillary dermis. Other limitations include the fact that expensive equipment and extensive training are required to assess RCM images properly. In addition, the procedure can be time-intensive, with the imaging time ranging from a few minutes to 45 minutes depending on the size and site of the target lesion.

### 2.1. Basic Principles

RCM has a near-infrared low-power laser light source (830 nm) that is directed toward the target skin and is reflected back toward the device [8,11]. The reflected light then passes through a pinhole that filters out any light, not from the targeted focal area. Each skin component has its own refractive index. RCM utilizes this index to create black and white images parallel to the skin surface from the reflected light [12]. Melanin and keratin have high refractive indexes and appear as bright white, whereas other components with low refractive indexes appear dark [12]. RCM can provide a lateral resolution of 0.5–1 μm and a penetration depth of 200–300 μm [8,11].

### 2.2. Clinical Applications for Specific Skin Cancers

#### 2.2.1. Squamous Cell Carcinoma

A systematic review of 25 studies reported that the sensitivity and specificity of RCM for the diagnosis of squamous cell carcinoma (SCC), SCC in situ, and keratoacanthoma were 79–100% and 78–100%, respectively [13]. The wide ranges in sensitivity and specificity may indicate that the diagnostic utility of RCM for these conditions is limited, which is partially demonstrated by the hyperkeratotic nature of SCC and the restricted penetration depth of RCM. RCM may be useful in monitoring actinic keratoses (AKs), which are pre-SCC lesions, after treatment [14,15,16].

#### 2.2.2. Basal Cell Carcinoma

Basal cell carcinoma (BCC) can be reliably detected by RCM with high sensitivity and specificity. A systematic review of RCM for the diagnosis of BCC reported a sensitivity of 97% and specificity of 93% [17]. In addition, BCC subtypes can be differentiated using RCM, which can be beneficial for the selection of optimal treatment [18]. However, the outcome of a recent randomized controlled multicenter study does not support the routine use of RCM as a replacement of biopsies in patients with a clinically suspected BCC [19]. In the study, biopsy outperformed RCM in diagnosing and differentiating subtypes of BCC [19]. The use of RCM for the detection of clinically invisible residual BCC after a biopsy has been studied [20]. This study reported that RCM could detect residual BCC with a sensitivity of 92.8% and specificity of 68.4% [20]. Previous studies demonstrated that RCM is also useful for evaluating the treatment response after non-surgical therapies for BCC, including photodynamic therapy, radiation therapy, and oral hedgehog inhibitors [21,22,23].

#### 2.2.3. Melanoma

A systematic review of 909 lesions reported that RCM had a sensitivity of 93% and specificity of 76% for the diagnosis of melanoma [24]. A more recent and larger systematic review of 7352 lesions reported a pooled sensitivity of 92% and specificity of 70% for the diagnosis of melanoma [25]. In the study, the diagnostic accuracy of RCM remained high regardless of the lesion type [25]. RCM was shown to be useful for the detection of amelanotic melanoma with poor dermoscopic features [26,27]. The use of RCM can also reduce the number of unnecessary biopsies. Studies have shown that the number of biopsies needed for the diagnosis of melanoma was reduced by up to 53% when RCM was combined with dermoscopy compared with the use of dermoscopy alone [28,29,30]. The use of RCM for planning and evaluating surgical margins for lentigo maligna (LM) has been studied. RCM was found to be superior to clinical and dermoscopic evaluation for the prediction of negative margins before surgical treatment for LM [31]. RCM may be useful in monitoring noninvasive therapy for melanoma and postoperative surveillance of melanoma recurrence. RCM showed that the number of atypical melanocytes was decreased after imiquimod therapy for LM [32]. It has been demonstrated that RCM can be effectively used to determine the nature of pigmentation around surgical scars [33]. Furthermore, the sensitivity of RCM for evaluating melanoma recurrence is higher than that of dermoscopy [34].

## 3. Optical Coherence Tomography

OCT provides real-time cross-sectional and en-face images of the tissue [35,36,37]. The resolution of OCT is insufficient for distinguishing cellular structures; however, it is sufficient for capturing structural changes. Although the resolution of OCT is lower than that of RCM, OCT can be used to evaluate more profound depths than RCM [35,36]. Further, it does not require preparation of the area under examination and can provide real-time images in less than a minute. An area of 6 × 6 mm^2^ can be covered automatically [38]. Several modified or enhanced OCT devices have been developed for specific purposes: Fourier domain-OCT (FD-OCT) detects the spectral components of the light source either with a spectrometer (spectral-domain [SD]-OCT) or a wavelength-swept laser (swept-source OCT). Compared with conventional OCT, FD-OCT can provide higher detection speed and sensitivity [39]. High-definition OCT (HD-OCT) has enhanced resolution; thus, it provides details of cellular structures [40]. Similar to a Doppler ultrasound system, angiographic OCT or dynamic OCT can be used to detect blood flow and achieve high-resolution images of the microvascular structures [41,42]. Line-field confocal OCT is a newly developed technique that allows imaging at a greater resolution than other types of OCT [43,44]. OCT has been applied for the diagnosis and tumor margin assessment of various types of skin cancer, including NMSCs and melanomas [36,45,46]. Although OCT has a high sensitivity for the diagnosis of NMSCs, its relatively lower specificity may lead to over-diagnosis [47,48]. Its inability to capture cellular details can result in uncertainty in discriminating between BCC and AK [48]. Cases of amelanotic melanoma misinterpreted as BCC by OCT have been reported [49]. Scars on the area of evaluation can prevent proper visualization of the underlying structures [35]. Similar to RCM, OCT requires expensive devices and extensive training for image interpretation [50].

### 3.1. Basic Principles

Near-infrared and infrared radiation (800–1300 nm) are used in OCT. The time delay and intensity of reflected echoes are measured using interferometry techniques, in which a comparison is made between back-scattered light from the target area and that from a reference mirror [36]. The contrast of the grayscale image is derived from the differences in refractive indexes of the skin components [51]. The depth of penetration is determined by the wavelength of the radiation, and the OCT device generally provides a scan depth of 2 mm, lateral resolution of 7.5 μm, and vertical resolution of 5–10 μm [35,50,52].

### 3.2. Clinical Applications for Specific Skin Cancers

#### 3.2.1. Squamous Cell Carcinoma

Generally, tumors of keratinocytes are not good candidates for OCT evaluation because the hyperkeratotic epidermis of those lesions prevents OCT from evaluating deeper layers. Although a meta-analysis reported high pooled sensitivity and specificity of OCT for the diagnosis of SCC [53], additional clinical studies are required to evaluate the utility of OCT for the diagnosis of SCC [54]. OCT has been studied for its usefulness in monitoring AK after treatment with various modalities, including ingenol mebutate, cryotherapy, laser ablation, and photodynamic therapy [55,56,57,58]. Due to its ability to detect subclinical AKs, OCT has a potential role in this clinical setting. HD-OCT and angiographic OCT might be helpful in differentiating AK from SCC [56,59]. The characteristic vascular features of AK, SCC in situ, and SCC were identified using angiographic OCT [60].

#### 3.2.2. Basal Cell Carcinoma

BCC is the skin cancer that is most widely studied using OCT. A multicenter, prospective study reported that the sensitivity and specificity of OCT for the diagnosis of BCC were 95.7% and 75.3%, respectively [38]. The diagnostic accuracy also increased from 65.8% for clinical examination alone to 87.4% for the combination of clinical examination and OCT [38]. Similarly, in a recent prospective study of 250 lesions, the specificity for the diagnosis of BCC increased from 47.5% to 76.8% when the clinical examination was supplemented by OCT evaluation, with a comparable sensitivity [61]. In another multicenter prospective study, OCT was shown to have significantly higher sensitivity and specificity for the diagnosis of BCC than clinical or dermoscopic evaluation [62]. In the study, the sensitivity and specificity of OCT were 92.9% and 80%, respectively, whereas the sensitivity and specificity of dermoscopy were 78.6% and 55.6%, respectively [62]. The authors determined that more than 35% of patients could avoid unnecessary biopsy with additional OCT evaluation [62]. In a previous study, experienced OCT users showed sensitivity values of 86–95% and specificity values of 81–98%, indicating that well-trained users are needed to perform this procedure [48]. A systematic review of 901 lesions from 31 studies reported an overall sensitivity of 89.3% and specificity of 60.3% for the diagnosis of BCC [63]. In the study, FD-OCT exhibited a higher diagnostic accuracy than conventional OCT [63]. These studies supported the adjunct role of OCT for the diagnosis of BCC. Besides this diagnostic role, OCT may be used to assess tumor depth and margin, which ultimately can help physicians select an optimal and individualized treatment option. The tumor depths of BCC measured using OCT were highly correlated with those measured during histological examinations [64]. OCT showed a potential to refine the surgical margin in Mohs micrographic surgery and to reduce the number of staged excisions required [45]. The treatment response after various modalities such as photodynamic therapy, topical therapy, laser, and oral vismodegib has been previously evaluated using OCT [55,65,66,67,68]. These studies found that OCT was superior to clinical examination alone for the detection of residual lesions. Due to its high resolution, HD-OCT may have higher specificity than conventional OCT for the diagnosis of BCC [69]. Although the subtypes of BCC were not readily discriminated using OCT, HD-OCT or angiographic OCT may show potential for differentiating among BCC subtypes and evaluating the aggressiveness of BCCs [41,70]. Preliminary data suggested that vascular morphology is correlated with the subtype of BCC [70].

#### 3.2.3. Melanoma

Melanocytic lesions have not been readily assessed using OCT, although some preliminary studies suggested the potential role of OCT for the diagnosis of melanoma [71,72]. A study reported that the sensitivity and specificity of HD-OCT for the diagnosis of melanoma were 74.1% and 92.4%, respectively [72]. In the study, HD-OCT showed high false-negative rates for thin melanomas and high false-positive rates for dysplastic nevi [72]. The potential role of angiographic OCT in the diagnosis of melanoma is noteworthy considering that early changes in vascular structures of melanoma can be detected using angiographic OCT [42]. In addition, the vascular density and morphology were shown to be correlated with the Breslow index and the stage of melanoma [73,74].

## 4. High-Frequency Ultrasound

Ultrasounds are a widely available imaging tool in general medicine. In the field of dermatology, HFUS with frequencies greater than 15 MHz can be optimally used to evaluate the skin and related structures [75]. The depth of penetration of ultrasounds is more than those of the previously mentioned diagnostic modalities and ranged from 4 mm to 7 mm depending on the frequency of ultrasounds [76]. Due to its noninvasive, time-efficient, and cost-effective characteristics, HFUS has been widely used for the diagnosis of various skin diseases, including skin cancer [77]. It can also be used in hard-to-process areas such as the inner ear and interdigital spaces [75]. However, HFUS does not provide sufficient resolution to evaluate cellular details, which limits its role in making a conclusive diagnosis of skin cancer. Instead, HFUS can be utilized as a reliable tool for evaluating the tumor size, depth, and location, which can lead to an optimal therapeutic decision [77,78,79,80,81]. HFUS can also be used to determine the treatment response and recurrence of skin tumors [76]. Overall low resolution, lack of functional contrast, operator-dependent image acquisition, and image quality are the limitations of HFUS [76,82]. The tumor depths measured using HFUS may not be precisely identical to those measured by histological examination because the tip of HFUS can actually compress the lesion, making it shallower. Moreover, histological specimens undergo processing, which affects the tumor thickness [77]. Tumor overestimation can occur because inflammatory infiltrates around the tumor are sometimes indistinguishable from tumor invasion by HFUS [83].

### 4.1. Basic Principles

The probe that generates ultrasounds is placed on the skin surface. Different reflections of sound waves are made depending on the impedance differences of the skin structures. These reflected waves are received by the probe and processed by a computer that generates a grayscale image [76]. The brightness of the image is dependent on the intensity of the reflected waves. High-intensity echoes create white or hyperechoic images, whereas low-intensity echoes create gray or hypoechoic images. Keratin and collagen determine the hyperechogenicity of the epidermis and dermis, respectively. In deeper layers, fascia and connective tissues are hyperechoic, whereas fatty tissues are hypoechoic [84]. The frequency of HFUS is proportional to the resolution of the image and inversely proportional to the penetration depth of sound waves. HFUS with a frequency of 20 MHz can provide a resolution of 50–200 μm with a penetrating depth of 6–7 mm. HFUS with a frequency of 50 MHz can provide a higher resolution of 39–120 μm with a lower penetrating depth of 4 mm [76].

### 4.2. Clinical Applications for Specific Skin Cancers

#### 4.2.1. Squamous Cell Carcinoma

A Cochrane systematic review published in 2018 did not identify any previous study on the diagnostic value of HFUS for SCC [85]. A recent retrospective study analyzed the HFUS features of AK, SCC in situ, and SCC. Based on these features, the study suggested that HFUS has acceptable diagnostic performance for AK, SCC in situ, and SCC (sensitivity of 85.3–92.3% and specificity of 73.6–88.0%) [86]. Another study that included 4338 skin lesions reported that HFUS did not demonstrate a significant diagnostic ability for malignant lesions [75]. HFUS has been used to differentiate between SCC and SCC in situ [76].

#### 4.2.2. Basal Cell Carcinoma

Only preliminary data on the utility of HFUS for the diagnosis of BCC have been reported [85]. HFUS may be utilized to rule out infiltrative BCCs and diagnose superficial BCCs. Tumor margins and subtypes of large tumors can be assessed using HFUS [80,87]. In a previous study, the tumor depth indexes of 18 BCC lesions were assessed using HFUS. The study found a strong correlation (correlation coefficient of 98.4%) between the ultrasonographic index and the histological index; however, the values for tumor depth obtained using HFUS were slightly lower than those obtained using histological examinations [77].

#### 4.2.3. Melanoma

Differentiating melanomas from melanocytic nevi using HFUS is challenging. HFUS can be combined with Color Doppler to enhance diagnostic accuracy by examining the tumoral vasculature [88]. In a previous study, Color Doppler showed a specificity of 100% at a sensitivity of 34% in discriminating melanoma from pigmented skin lesions [89]. The assessment of tumor depth using HFUS has been reported to be reliable [79]. In one study, the intraclass correlation coefficient between tumor depth measured using HFUS and that measured using histopathology was 0.807 (95% confidence interval, 0.703–0.877), with high intra- and inter-reproducibility [79]. Another study that included eight superficial spreading melanomas and 20 nodular melanomas (NMs) found that the correlation between the tumor depth index measured using HFUS and that measured using histopathology was greater than 98%. However, HFUS offered a slightly higher index for superficial spreading melanoma and a lower value for NM than histology [77]. 

## 5. Electrical Impedance Spectroscopy

EIS was developed as an adjunct diagnostic tool for skin cancer. Although it cannot provide a definitive diagnosis of skin cancer, it can provide information that is helpful for making the decision regarding whether to perform an invasive biopsy [90]. By comparing the electrical properties of skin lesions to those of normal skin, EIS generates scores from 0 to 10 [91]. Generally, skin lesions with scores of >3 can be considered malignant, and such lesions need to be biopsied. A handheld probe covering an area of 5 × 5 mm^2^ is applied to the skin, and a painless electrical current is generated and detected by the probe. Each measurement takes less than 10 s, and multiple measurements may be required to cover large lesions [90,92]. EIS can be used for the detection of various types of skin cancer, including NMSC and melanoma, without the requirement of extensive training [90,92]. Furthermore, it can be utilized to monitor doubtful lesions over time [93]. EIS shows high sensitivity for the detection of skin cancer at the expense of specificity. False-negative results may be generated in small lesions with a diameter of <2 mm or lesions with low cellularity [90]. Another limitation is that EIS classifies a large proportion of seborrheic keratosis as skin cancer [90,92]. As with most of the other techniques, skin lesions with ulceration, inflammation, scars, or foreign materials such as tattoos and splinters are not readily assessable using EIS. In addition, skin lesions that are located on the palms; soles; scalp with hair; bony, curved, or mucosal areas; and soft areas such as the abdomen cannot be properly assessed using EIS [92,93].

### 5.1. Basic Principles

An evoked electrical current is applied and the amplitude of the current through the skin lesion is measured and converted into digital signals by EIS. The data from skin lesions and reference areas are compared, and a computerized algorithm generates scores ranging from 0 to 10. The score is correlated with the stage and cellular atypia of a lesion. The malignant transformation of cells changes the electrical properties of human tissues, which are associated with the size, shape, and membrane structure of cells and are detected using EIS. To increase the accuracy and stability of measurement, disposable and skin-penetrating micro-electrodes are employed. The electrical properties of the skin are measured at different frequencies and at different depths [94]. The penetration depth of the electrical current is up to 2.5 mm [92].

### 5.2. Clinical Applications for Specific Skin Cancers

#### 5.2.1. Non-Melanoma Skin Cancer

EIS does not seem to be capable of differentiating among various NMSCs; however, it appears to have high sensitivity and low-to-moderate specificity for the detection of NMSCs. A study that included 48 BCCs and 7 SCCs reported a sensitivity of EIS of 100% [90]. In another study, the sensitivity of EIS for the detection of NMSCs was 71% [95]. A recent pilot study of 138 malignant lesions reported that the sensitivity and specificity of EIS for the detection of NMSCs were 94.2% and 41.9%, respectively [92].

#### 5.2.2. Melanoma

Melanoma is the most widely studied type of skin cancer using EIS. A multicenter, prospective, blinded clinical trial reported that EIS had a sensitivity of 96.6% and specificity of 34.4% for the detection of melanoma. These results are promising because the cohort mostly comprised in situ and early melanomas [90]. Another study compared the diagnostic accuracy of clinical assessment alone with that of clinical assessment and EIS. After the addition of EIS, the sensitivity for melanoma diagnosis increased from 81% to 98%, whereas the specificity decreased from 84% to 55% [95]. However, in a recent study, when both EIS score and clinical assessment were used to rule out melanoma, both the sensitivity (95.2%) and specificity (58.6%) were significantly higher than those of only clinical assessment (sensitivity of 80.7% and specificity of 50.4%) [96]. In addition, the diagnostic accuracy improved with the additional EIS examination, which resulted in a reduction in the number of biopsies of benign lesions [96]. Similarly, including EIS examination in the clinical decision-making process significantly decreased the number needed to biopsy from 6.3 to 5.3 in another recent study [97]. Melanomas present in the extremities and trunk were better detected using EIS (sensitivity of 91% and specificity of 64%) than those located at the head and neck region [95]. There have been conflicting data on the performance of EIS depending on the thickness of the melanoma. A study reported that EIS had higher sensitivity for thin or in situ melanomas (100%) than for thicker melanomas (81%) [95]; however, another study reported higher sensitivity for more invasive melanomas (100%) than for in situ melanomas (88%) [98].

## 6. Pigmented Lesion Assay

PLA is a noninvasive adhesive patch-based gene expression test [99,100]. This technology is unique in that it analyzes the genetic profiling of suspicious pigmented lesions rather than microscopic structures to detect malignant melanoma. Four round adhesive patches are applied to the pigmented lesion and genetic material is isolated from the patches [101]. Previously, a classification system based on 17 genes was proposed [99]. Using an optimized algorithm, the system showed high sensitivity and specificity for the detection of melanoma. Thereafter, a simplified system using only two genes: long intergenic non-protein coding RNA 518 (*LINC00518*) and preferentially expressed antigen in melanoma (*PRAME*), has been developed and validated [100,102,103]. This simplified system does not need a complex algorithm; thus, it is cost- and time-efficient. The performance of this two-gene assay was similar to the previous system based on 17 genes. Considering the inter-rater variability in histopathological examinations for pigmented lesions, especially lesions with borderline malignant characteristics, PLA may be used to assist the accurate diagnosis of histopathologically uncertain lesions [104]. PLA may not be utilized in certain areas, including the mucous membrane, soles, palms, and nails [100,105]. There is a possibility of insufficient tissue collection for PLA testing, which is reported to occur in up to 14% of test attempts [100]. Although *PRAME* expression is frequently detected in melanomas, certain subtypes, including desmoplastic melanomas, show a lower frequency of this gene expression [106], limiting the use of PLA for the detection of these melanomas.

### 6.1. Basic Principles

Adhesive patches are used in PLA to collect genetic information from the stratum corneum. Malignant melanocytes have been proposed to activate keratinocytes to produce melanocytic mRNAs or disperse mRNA to the stratum corneum in a similar manner as pigment migration [99]. Total RNA is isolated from the adhesive patches and reverse transcribed to complementary DNA. Using quantitative real-time polymerase chain reaction, complementary DNA is used to analyze target gene expression [100]. The gene expression profiles of adhesive patches were found to be consistent with those of underlying formalin-fixed, paraffin-embedded samples of excised primary melanomas and melanomas metastasized to lymph nodes [100]. A positive result in PLA is highly suggestive of the presence of somatic mutations in genes such as *BRAF*, *NRAS*, and *TERT*, which play a role in melanoma development and progression [107].

### 6.2. Clinical Applications for Specific Skin Cancers

#### Melanoma

PLA has been reported to have high sensitivity and moderate-to-high specificity for the diagnosis of melanoma. A study comprising a cohort of 398 pigmented lesions reported that the sensitivity and specificity of PLA for the detection of melanoma were 91% and 69%, respectively [100]. Another study comprising 381 pigmented lesions reported an even higher sensitivity of 95% and specificity of 91% [102]. The reported high negative predictive value of PLA of >99% can prevent unnecessary invasive biopsy procedures [100]. The high negative predictive value of PLA was supported by a recent study. In the study, clinicians chose to monitor 98.2% of 743 PLA-negative cases for 12 months without biopsy procedures. None of the 13 lesions for which biopsy specimens were available were diagnosed as melanoma histopathologically [108]. Indeed, the impact of PLA outcomes on clinical decision-making was described in real-world setting studies. About 99% of PLA-negative cases were followed up without biopsy procedures, while 97% of PLA-positive cases underwent histopathological examination [108,109]. The incorporation of PLA in the decision to perform biopsy resulted in a decrease in the total number of biopsies and an increase in the rate of detection of early melanomas [105]. PLA decreased the biopsy ratio from 12.5 with clinical examination alone to 2.4 with additional PLA data [110]. With PLA, the number needed to biopsy was as low as 1.4–2.7 [102,110]. A decrease in the number of unnecessary biopsies can reduce the associated health care costs [103]. The usefulness of PLA in a patient with numerous atypical, pigmented lesions has also been described. In the study, PLA allowed for efficient screening of multiple atypical lesions simultaneously [111].

## 7. Raman Spectroscopy

RS is performed by applying a handheld probe on the skin and recording the Raman spectra of the molecules [112]. The probe emits near-infrared laser radiation that covers an area with a diameter of 3.5 mm. The internalized algorithm analyzes the spectral information, generating a predicted probability of the lesion being either benign or malignant [112,113]. Due to its long acquisition time, RS was originally used only in *ex vivo* tissue. With advances in technology, the in vivo application of RS has become possible, and its use for the diagnosis of various types of skin cancer has been investigated [113]. RS showed moderate-to-high sensitivity and specificity for the diagnosis of skin cancer [114]. These parameters may improve with advances in technology because the diagnostic accuracy of in vivo assessment was found to be lower than that of *ex vivo* assessment [114]. Besides the long acquisition time, the high cost and weak imaging signals are limitations of RS [112,113].

### 7.1. Basic Principles

RS is based on the Raman effect, a physical principle that refers to the minuscule change in energy between an incident and a scattered photon [112,113]. Chemical bonds of skin-constituting molecules are responsible for the change of energy specific to each targeted molecule. A near-infrared laser with a wavelength of 785 nm or 830 nm is used to generate photons. The changes in these photons’ energy in inelastic scattering by molecules in target tissues are detected using spectrometers [112,113]. The measurable depth is reported to range from 300 μm to 1 mm [115,116].

### 7.2. Clinical Applications for Specific Skin Cancers

#### NMSC and Melanoma

The sensitivity of RS can be elevated at the expense of specificity. In a study comprising 518 benign and malignant lesions, RS showed a specificity of 64% and a sensitivity of 90% when discriminating skin cancer plus AK from benign lesions [113]. The specificity decreased to 41% when the sensitivity level increased to 95% [113]. In the same study, melanoma was discriminated from benign pigmented lesions at a specificity of 68% and sensitivity of 90% [113]. The algorithm was improved to have a specificity of 75% at a sensitivity of 90% when differentiating between malignant and benign lesions [117]. A review of 12 studies that evaluated the use of RS for the diagnosis of various types of skin cancer reported a sensitivity of 69% and specificity of 85% for BCC, the sensitivity of 81% and specificity of 89% for SCC, and sensitivity of 93% and specificity of 96% for melanoma [114]. The accuracy of the proposed system with an RS device for the diagnosis of skin cancer was found to be higher than that of general practitioners and trainees and comparable to that of dermatologists [118]. The system had a sensitivity of 90% and a specificity of >32% for determining benignity [118]. Recently, RS was suggested to be a promising tool to assess surgical margins during Mohs micrographic surgery of BCC [119].

## 8. Conclusions and Future Perspectives

Diagnostic modalities for skin cancer that are noninvasive, reliable, accurate, objective, affordable, and both physician and patient-friendly have been sought. Among the candidates for an ideal adjunctive diagnostic method, the technologies discussed in this review have advantages and disadvantages (Table 1). They have limitations such as high cost, low specificity, and lack of efficiency. Another issue to consider is that as of this writing, most of them are not readily applicable to lesions with hyperkeratosis, ulcer, severe inflammation, or foreign bodies or lesions located on certain anatomic sites. Despite these barriers, they are constantly being improved or combined to overcome their limitations without compromising their advantages. The slow image acquisition processes of RCM and RS are improving with advances in technology. The RCM device is being improved to have a better range of motion with minimal artifacts. Software for RCM are also being developed to produce images with pseudo-colors that have a better correlation with the histopathological image [26]. The combination of RCM and OCT has demonstrated encouraging results by compensating the limitations of each device [120]. OCT has also been combined with multiphoton tomography to provide high cellular resolution [52]. Short-noise limited, supercontinuum-based OCT is also being developed, which generates images with higher contrast, better sensitivity, and improved penetration than previous OCT images [39]. Machine-based learning can be combined with RCM and RS to improve diagnostic accuracy without a need for extensive training of physicians [112,121]. 

In the era of COVID-19, teledermatology has become increasingly popular [122]. These emerging techniques for skin cancer diagnosis have the potential to be part of teledermatology in primary care settings. There is also the possibility that artificial intelligence could be combined with any of the emerging techniques and positively contribute to the widespread use of these technologies. Finally, to prevent the emerging techniques from remaining as merely experimental equipment employed in few special centers, more future large, multicenter, prospective clinical trials on the use of these techniques are needed. This can be achieved by constant interest on this issue not only by health providers, but the general population, patients, and health-related organizations of each country.

In conclusion, emerging devices that are capable of detecting different types of skin cancer at an early stage will become more readily available with the advancement of technology and offer greater benefits to medical practice in the field of skin cancer. Ultimately, this will actualize the optimal individualization of treatment for each patient. 

## Figures and Tables

**Table 1:** Summary of technologies for the detection of skin cancer.

Technology	Main Target Skin Cancer	Resolution	Evaluable Depth	Requirement of Extensive Training	Advantages	Limitations
Reflectance confocal microscopy	SCC (sensitivity 79–100%, specificity 78–100%) [13], BCC (sensitivity 97%, specificity 93%) [17], melanoma (sensitivity 92%, specificity 70%, 22–53% reduction of unnecessary excisions) [25,28,29,30]	0.5–1 μm (lateral)	200–300 μm	Yes	High sensitivity and specificity Provides cellular resolution Can decrease the number of unnecessary biopsies Allows tumor margin mapping Enables evaluation of treatment response Enables surveillance for tumor recurrence Useful for diagnosis of amelanotic melanoma	Limited depth of penetration Slow image acquisition time Hyperkeratotic lesions are difficult to assess Advanced training is required High cost of device
Optical coherence tomography	BCC (sensitivity 89–96%, specificity 60–98%, >35% reduction of unnecessary biopsies) [38,48,61,62,63]	7.5 μm (lateral), 5–10 μm (vertical)	2 mm	Yes	High sensitivity and specificity Can decrease the number of unnecessary biopsies Can assess tumor margins and depth Enables evaluation of response Rapid image acquisition	Lack of cellular detail Amelanotic melanoma can be misdiagnosed as BCC Hyperkeratotic lesions are difficult to assess Advanced training is required High cost of device
High-frequency ultrasound	BCC, melanoma	50–200 μm	4–7 mm	Yes	Deep penetration depth Rapid image acquisition	Low resolution Operator-dependent image quality Overestimation of tumor size Advanced training is required
Electrical impedance spectroscopy	NMSC (sensitivity 71–100%, specificity 42%) [90,92,95], melanoma (sensitivity 95–98%, specificity 34–59%, 16% reduction of unnecessary biopsies) [90,95,96,97]	-	2.5 mm	No	High sensitivity Can assist clinical decisions to biopsy	Low specificity False-positive results may occur with seborrheic keratosis
Pigmented lesion assay	Melanoma (sensitivity 91–95%, specificity 69–91%, 81% reduction of biopsy ratio) [100,102,110]	-	-	No	High sensitivity with moderate-to-high specificity May provide diagnostic information Can decrease the number of unnecessary biopsies	May not be used in certain anatomical areas Repeated sampling can be required Limited value on certain subtypes of melanoma
Raman spectroscopy	NMSC (sensitivity 69–81%, specificity 85–89%) [114], melanoma (sensitivity 90–93%, specificity 68–96%) [113,114]	-	300 μm–1 mm	No	High specificity	High cost of device Slow image acquisition time Fewer clinical studies than other technologies

## Data Availability

Not applicable.
